# Tuberculosis Testing and Latent Tuberculosis Infection Treatment Practices Among Health Care Providers — United States, 2020–2022

**DOI:** 10.15585/mmwr.mm7244a2

**Published:** 2023-11-03

**Authors:** Elise Caruso, Joan M. Mangan, Allison Maiuri, Beth Bouwkamp, Nickolas DeLuca

**Affiliations:** ^1^Division of Tuberculosis Elimination, National Center for HIV, Viral Hepatitis, STD, and TB Prevention, CDC; ^2^Oak Ridge Institute for Science and Education, Oak Ridge, Tennessee.

**Summary**

**What is already known about this topic?**

CDC recommends testing persons at increased risk for tuberculosis (TB) infection as part of routine health care using TB blood tests, when possible, and if a diagnosis of latent TB infection (LTBI) is made, prescribing a short-course treatment regimen. In 2022, approximately three quarters of reported U.S. TB cases occurred among non–U.S.-born persons.

**What is added by this report?**

Among 3,647 health care providers, approximately one half (53%) reported routinely testing non–U.S.-born patients for TB. More than one half (59%) reported prescribing any LTBI treatment; 33% reported prescribing short-course regimens. In addition, 41% referred patients to a health department for treatment.

**What are the implications for public health practice?**

Identifying and overcoming barriers to recommended testing and treatment is important to prevent disease and achieve TB elimination goals.

## Abstract

CDC recommends testing persons at increased risk for tuberculosis (TB) infection as part of routine health care, using TB blood tests, when possible, and, if a diagnosis of latent TB infection (LTBI) is made, prescribing a rifamycin-based, 3- or 4-month treatment regimen (short-course) to prevent the development of TB disease. In 2022, approximately three quarters (73%) of reported TB cases in the United States occurred among non–U.S.-born persons. To assess TB-related practices among health care providers (HCPs) in the United States, CDC analyzed data from the 2020–2022 Porter Novelli DocStyles surveys. Approximately one half (53.3%) of HCPs reported routinely testing non–U.S.-born patients for TB, and of those who did, 35.7% exclusively ordered recommended blood tests, 44.2% exclusively ordered skin tests, and 20.2% ordered TB skin tests and blood tests. One third (33.0%) of HCPs reported prescribing recommended short-course LTBI treatment regimens, and 4.0% reported doing none of the treatment practices available for patients with LTBI (i.e., prescribing short-course regimens, longer course regimens, or referring patients to a health department). Further efforts are needed to identify and overcome barriers for providers to test for and treat persons at risk for TB. 

## Introduction

CDC estimates that up to 13 million persons in the United States have latent tuberculosis infection (LTBI) (*1*). Approximately 5%–10% of persons with LTBI in the United States who remain untreated will develop tuberculosis (TB) disease at some point in their lifetime. TB disease is infectious and can be fatal. In 2022, approximately three quarters (73%) of reported TB cases in the United States occurred among non–U.S.-born persons (*2*). The most common countries of birth among non–U.S.-born persons with TB have been China India, Mexico, Philippines, and Vietnam.* Efforts to eliminate TB in the United States include finding and treating persons with TB disease, expanding LTBI testing and treatment to prevent progression to TB disease, and addressing disparities among groups disproportionately experiencing impacts of TB. Since 1992, TB cases have generally decreased in the United States; however, ongoing TB prevention and control efforts are needed to continue this trend and achieve TB elimination in the United States (<1 case per million persons annually) (*2*).

Persons who were born in countries where TB disease is common are at increased risk for TB infection (*3*). In addition, many persons born outside the United States have received the Bacille Calmette-Guérin (BCG) TB vaccine. This vaccine is often given to infants and small children in countries where TB is common to decrease the risk for childhood TB meningitis and disseminated disease; however, it is not thought to prevent pulmonary TB disease in adolescents and adults, and protection wanes over time (*4*). Having previously received the BCG vaccine can cause a false-positive reaction to TB skin tests, leading to falsely diagnosing TB infection or conversely, misattributing a positive TB test result to childhood BCG vaccination, even though the patient does have TB infection (*5*). TB blood tests are not affected by previous BCG vaccination. When possible, CDC recommends that health care providers (HCPs) test persons at risk for TB using TB blood tests (interferon-gamma release assays), and if a diagnosis of LTBI is made, prescribe a short-course LTBI treatment regimen in preference to longer course 6- or 9-month isoniazid monotherapy (*2*,*6*). Persons at increased risk for TB infection should be tested for TB infection as part of routine health care (*6*). TB-related questions were added to the Porter Novelli DocStyles survey to assess HCP testing and treatment practices.

## Methods

### Data Collection

Porter Novelli conducts online surveys of U.S. HCPs. TB questions were included in the 2020, 2021, and 2022 DocStyles annual fall surveys. Each year Porter Novelli sets quotas to collect completed surveys from 1,000 primary care physicians (i.e., family practitioners and internists) and 250 each of obstetricians/gynecologists, pediatricians, and nurse practitioners or physician assistants. Respondents must practice in the United States; actively see patients; work in an individual, group, or inpatient or hospital practice; and have been practicing for ≥3 years. Respondents were asked, “Do you routinely test non–U.S.-born patients for tuberculosis (TB)?” and instructed to select one of the following response options: “Yes, with a TB blood test,” “Yes, with a TB skin test,” “Yes, with a TB blood test and skin test,” “No, I do not regularly test for TB,” “I refer patients to the health department,” and “Prefer not to answer.” Respondents were also asked to select all of the following LTBI treatment regimens they prescribe: “Isoniazid & Rifapentine - 3 months (3HP),” “Rifampin - 4 months (4R),” “Isoniazid & Rifampin - 3 months (3HR),” “Isoniazid - 6 months (6H),” “Isoniazid - 9 months (9H),” “I refer patients to the health department,” and “None of these.” Additional information on methods and response rates is available on the Porter Novelli website.^†^

### Data Analysis

Data from the three survey years were combined by retaining variables that were consistent among the years and, for respondents who participated in more than 1 year, respondents’ most recent survey participation year of data. DocStyles respondents who responded “prefer not to answer” to either TB question were excluded (47), and obstetricians/gynecologists were excluded because they were not asked questions about TB (563). Data from 3,647 DocStyles respondents for 2020–2022 were retained and analyzed. Percentages were calculated for demographic characteristics and TB-related variables. Pearson’s chi-square and Fisher’s exact tests were performed to examine associations between demographic characteristics and responses to TB questions. Associations were considered statistically significant if p-values were <0.05. For significant chi-square associations, post-hoc calculations of adjusted standardized residuals were performed. Bonferroni corrections were applied to chi-square p-values and adjusted standardized residual critical values to reduce the likelihood of type I error (*7*). Analyses were conducted using SPSS software (version 27; IBM). This activity was reviewed by CDC, deemed not research, and was conducted consistent with applicable federal law and CDC policy.^§^

## Results

### TB Testing Practices

Among 3,647 respondents, approximately one half (1,945; 53.3%) reported routinely testing non–U.S.-born patients for TB. A total of 1,446 (39.6%) reported not regularly testing non–U.S.-born patients for TB, and 256 (7.0%) reported referring non–U.S.-born patients to a health department for TB testing (Table 1). The groups with the highest proportion reporting that they routinely test non–U.S.-born patients for TB were pediatricians (63.1%), providers aged >55 years (60.3%), those in practice for >25 years (60.4%), and those practicing in group outpatient settings (56.7%). The HCPs with the highest percentage reporting that they did not regularly test non–U.S.-born patients for TB (50.2%) were those working in inpatient or hospital practices. The HCP groups with the highest percentage reporting referring non–U.S.-born patients to a health department for TB testing were nurse practitioners (14.1%) and those working in rural settings (12.8%). Among the 1,945 providers who reported regularly testing non–U.S.-born patients for TB, 859 (44.2%) reported using TB skin tests, 694 (35.7%) reported using TB blood tests, and 392 (20.2%) reported using TB skin tests and blood tests (Table 2). Among the 859 respondents who reported using a TB skin test, this practice was more prevalent among those who worked in rural settings (59.6% of respondents practicing in rural settings).

**TABLE 1:** Type of tuberculosis testing practices* for non–U.S.-born patients, by health care provider characteristics (N = 3,647) — DocStyles survey, United States, 2020–2022

Characteristic	No. (column %)	Testing practice, no. (row %)			p-value^§^
	Total	Any type of TB test^†^	Do not regularly test for TB	Refer patients to a health department	
**Overall**	**3,647 (100.0)**	**1,945 (53.3)**	**1,446 (39.6)**	**256 (7.0)**	—
**Specialty**					
Family practitioner	**1,073 (29.4)**	606 (56.5)	403 (37.6)	64 (6.0)	<0.001^¶^
Internist	**1,286 (35.3)**	670 (52.1)	548 (42.6)	68 (5.3)	
Nurse practitioner	**305 (8.4)**	127 (41.6)**	135 (44.3)	43 (14.1)**^††^**	
Pediatrician	**629 (17.2)**	397 (63.1)**^††^**	192 (30.5)**	40 (6.4)	
Physician assistant	**354 (9.7)**	145 (41.0)**	168 (47.5)	41 (11.6)	
**Gender^§§^**					
Female	**1,518 (41.6)**	737 (48.6)	655 (43.1)	126 (8.3)	<0.001^¶,¶¶^
Male	**2,115 (58.0)**	1,198 (56.6)	788 (37.3)	129 (6.1)	
Other	**14 (0.4)**	10 (71.4)	3 (21.4)	1 (7.1)	
**Age group, yrs**					
25–40	**1,328 (36.4)**	653 (49.2)**	571 (43.0)	104 (7.8)	<0.001^¶^
41–55	**1,500 (41.1)**	798 (53.2)	606 (40.4)	96 (6.4)	
>55	**819 (22.5)**	494 (60.3)**^††^**	269 (32.8)	56 (6.8)	
**Yrs in practice**					
3–10	**1,291 (35.4)**	627 (48.6)**	559 (43.3)	105 (8.1)	<0.001^¶^
11–25	**1,727 (47.4)**	938 (54.3)	681 (39.4)	108 (6.3)	
>25	**629 (17.2)**	380 (60.4)**^††^**	206 (32.8)^¶^	43 (6.8)	
U.S. Census Bureau region***					
Northeast	**780 (21.4)**	429 (55.0)	300 (38.5)	51 (6.5)	0.364
Midwest	**959 (26.3)**	475 (49.5)	413 (43.1)	71 (7.4)	
South	**1,131 (31.0)**	590 (52.2)	448 (39.6)	93 (8.2)	
West	**777 (21.3)**	451 (58.0)	285 (36.7)	41 (5.3)	
**Urban-rural status^†††^**					
Urban	**1,387 (38.0)**	779 (56.2)	523 (37.7)	85 (6.1)	<0.001^¶^
Suburban	**1,868 (51.2)**	995 (53.3)	752 (40.3)	121 (6.5)	
Rural	**392 (10.7)**	171 (43.6)**	171 (43.6)	50 (12.8)**^††^**	
**Work setting**					
Individual outpatient practice	**573 (15.7)**	301 (52.5)	215 (37.5)	57 (9.9)	<0.001^¶^
Group outpatient practice	**2,436 (66.8)**	1,381 (56.7)**^††^**	911 (37.4)**	144 (5.9)**	
Inpatient practice	**638 (17.5)**	263 (41.2)**	320 (50.2)**^††^**	55 (8.6)	
**Approximate patient household income**					
<$25,000	**234 (6.4)**	121 (51.7)	94 (40.2)	19 (8.1)	0.999
$25,000–$49,999	**895 (24.5)**	460 (51.4)	362 (40.4)	73 (8.2)	
$50,000–$99,999	**1,436 (39.4)**	743 (51.7)	597 (41.6)	96 (6.7)	
$100,000–$249,999	**749 (20.5)**	438 (58.5)	258 (34.4)	53 (7.1)	
≥$250,000	**333 (9.1)**	183 (55.0)	135 (40.5)	15 (4.5)	

**Abbreviation:** TB = tuberculosis.

* Respondents were asked to select one response to the question “Do you routinely test non–U.S.-born patients for tuberculosis (TB)?” Those who selected “Prefer not to answer” were removed from the DocStyles sample (47). Percentages might not sum to 100 because of rounding.

^†^ Responses of “TB skin test,” “TB blood test,” or “TB skin test and blood test” were grouped into the category of “Any type of TB test” for analysis.

^§^ Associations between provider characteristics and responses to TB testing questions were calculated using Pearson’s chi-square tests with Bonferroni-corrected p-values unless otherwise indicated, in which case Fisher’s exact test was used.

^¶^ Value is statistically significant at p<0.05.

** Adjusted standardized residual ≤−3.6, indicating significantly less than expected cell value.

**^††^** Adjusted standardized residual ≥3.6, indicating significantly greater than expected cell value.

^§§^ The “Other” option for gender was not available in the 2020 survey.

^¶¶^ Because of small cell sizes, significance was calculated using the Monte Carlo approximation for Fisher’s exact test (based on 10,000 sampled tables at a 99% CI). Adjusted standardized residuals were not calculated.

*** https://www2.census.gov/geo/pdfs/maps-data/maps/reference/us_regdiv.pdf

**^†††^** Determined by the question, “How would you describe the community where you primarily work?”

**TABLE 2:** Type of test used by health care providers who reported any tuberculosis testing of non–U.S.-born patients, by health care provider characteristics (N = 1,945) — DocStyles survey, United States, 2020–2022

Characteristic	Total (column %)	Type of test used, no. (row %)*			p-value^†^
		TB skin test	TB blood test	TB skin test and blood test	
**Overall**	**1,945 (100.0)**	**859 (44.2)**	**694 (35.7)**	**392 (20.2)**	—
**Specialty**					
Family practitioner	**606 (31.2)**	279 (46.0)	206 (34.0)	121 (20.0)	0.164
Internist	**670 (34.4)**	254 (37.9)	274 (40.9)	142 (21.2)	
Nurse practitioner	**127 (6.5)**	62 (48.8)	46 (36.2)	19 (15.0)	
Pediatrician	**397 (20.4)**	196 (49.4)	116 (29.2)	85 (21.4)	
Physician assistant	**145 (7.5)**	68 (46.9)	52 (35.9)	25 (17.2)	
**Gender^§^**					
Female	**737 (37.9)**	344 (46.7)	239 (32.4)	154 (20.9)	0.150^¶^
Male	**1,198 (61.6)**	511 (42.7)	452 (37.7)	235 (19.6)	
Other	**10 (0.5)**	4 (40.0)	3 (30.0)	3 (30.0)	
**Age group, yrs**					
25–40	**653 (33.6)**	253 (38.7)	262 (40.1)	138 (21.1)	0.059
41–55	**798 (41.0)**	357 (44.7)	270 (33.8)	171 (21.4)	
>55	**494 (25.4)**	249 (50.4)	162 (32.8)	83 (16.8)	
**Yrs in practice**					
3–10	**627 (32.2)**	247 (39.4)	252 (40.2)	128 (20.4)	0.114
11–25	**938 (48.2)**	416 (44.3)	322 (34.3)	200 (21.3)	
>25	**380 (19.5)**	196 (51.6)	120 (31.6)	64 (16.8)	
U.S. Census Bureau region**					
Northeast	**429 (22.1)**	186 (43.4)	151 (35.2)	92 (21.4)	0.999
Midwest	**475 (24.4)**	213 (44.8)	178 (37.5)	84 (17.7)	
South	**590 (30.3)**	279 (47.3)	196 (33.2)	115 (19.5)	
West	**451 (23.2)**	181 (40.1)	169 (37.5)	101 (22.4)	
**Urban-rural status^††^**					
Urban	**779 (40.1)**	291 (37.4)^§§^	312 (40.1)	176 (22.6)	<0.001***
Suburban	**995 (51.2)**	466 (46.8)	342 (34.4)	187 (18.8)	
Rural	**171 (8.8)**	102 (59.6)^¶¶^	40 (23.4)	29 (17.0)	
**Work setting**					
Individual outpatient practice	**301 (15.5)**	142 (47.2)	104 (34.6)	55 (18.3)	0.999
Group outpatient practice	**1,381 (71.0)**	610 (44.2)	495 (35.8)	276 (20.0)	
Inpatient practice	**263 (13.5)**	107 (40.7)	95 (36.1)	61 (23.2)	
**Approximate patient household income**					
<$25,000	**121 (6.2)**	56 (46.3)	45 (37.2)	20 (16.5)	0.999
$25,000–$49,999	**460 (23.7)**	199 (43.3)	170 (37.0)	91 (19.8)	
$50,000–$99,999	**743 (38.2)**	351 (47.2)	256 (34.5)	136 (18.3)	
$100,000–$249,999	**438 (22.5)**	188 (42.9)	154 (35.2)	96 (21.9)	
≥$250,000	**183 (9.4)**	65 (35.5)	69 (37.7)	49 (26.8)	

**Abbreviation:** TB = tuberculosis.

* Respondents were asked to select one response to the question “Do you routinely test non–U.S.-born patients for tuberculosis (TB)?” Those who selected “Prefer not to answer” were removed from the DocStyles sample (47). Percentages might not sum to 100 because of rounding.

^†^ Associations between provider characteristics and responses to TB testing questions were calculated using Pearson’s chi-square tests with Bonferroni-corrected p-values unless otherwise indicated, in which case Fisher’s exact test was used.

**^§^** The “Other” option for gender was not available in the 2020 survey.

^¶^ Because of small cell sizes, significance was calculated using the Monte Carlo approximation for Fisher’s exact test (based on 10,000 sampled tables at a 99% CI). Adjusted standardized residuals were not calculated.

** https://www2.census.gov/geo/pdfs/maps-data/maps/reference/us_regdiv.pdf

^††^ Based on responses to the question, “How would you describe the community where you primarily work?”

^§§^ Adjusted standardized residual ≤−3.6, indicating significantly less than expected cell value.

^¶¶^ Adjusted standardized residual ≥3.6, indicating significantly greater than expected cell value.

*** Value is statistically significant at p<0.05.

### LTBI Treatment Practices

Among all 3,647 respondents, one third (1,203; 33.0%) reported prescribing recommended short-course regimens to treat LTBI, 1,349 (37.0%) reported prescribing longer course treatments, and 1,490 (40.9%) reported referring patients to a health department for LTBI treatment (Table 3) (responses were not mutually exclusive). More than one half (59.1%) reported prescribing any LTBI treatment (either short- or long-course regimens). By provider group characteristic, those who most commonly prescribed short-course treatment regimens included internists (41.1%), those practicing in urban settings (39.4%), and in inpatient or hospital practices (39.7%). The highest percentages of providers who reported referring patients to a health department for LTBI treatment were nurse practitioners (64.3%), physician assistants (60.7%), and those working in rural settings (56.4%). Among providers across all U.S. Census Bureau regions, those in the West reported the lowest prevalence of health department referrals for LTBI treatment (32.0%), but when offering treatment, these providers most often reported prescribing longer-course LTBI treatment regimens (43.6%). Overall, 9.6% of physician assistants reported doing “none of these” LTBI treatment practices, the highest prevalence among all specialty groups (2.2% family practitioner, 3.9% internist, 6.2% nurse practitioner, and 3.2% pediatrician).

**TABLE 3:** Latent tuberculosis infection treatment prescribing practices reported by health care providers, by health care provider characteristics (N = 3,647) — DocStyles survey, United States, 2020–2022

Characteristic	Treatment regimens for latent tuberculosis infection prescribed, no. (row %)*											
	Short course treatment regimen (3HP, 3HR, or 4R)		p-value^†^	Longer course treatment regimen (6H or 9H)		p-value^†^	Refer patients to a health department		p-value^†^	None of these		p-value^†^
	Yes	No		Yes	No		Yes	No		Yes	No	
**Overall**	**1,203 (33.0)**	**2,444 (67.0)**	—	**1,349 (37.0)**	**2,298 (63.0)**	—	**1,490 (40.9)**	**2,157 (59.1)**	—	**147 (4.0)**	**3,500 (96.0)**	—
**Specialty**												
Family practitioner	392 (36.5)	681 (63.5)	<0.001^§^	425 (39.6)	648 (60.4)	<0.001^§^	418 (39.0)	655 (61.0)	<0.001^§^	24 (2.2)^¶^	1,049 (97.8) **	<0.001^§^
Internist	529 (41.1)**	757 (58.9)^¶^		580 (45.1)**	706 (54.9)^¶^		378 (29.4)^¶^	908 (70.6)**		50 (3.9)	1,236 (96.1)	
Nurse practitioner	71 (23.3)^¶^	234 (76.7)**		45 (14.8)^¶^	260 (85.2)**		196 (64.3)**	109 (35.7)^¶^		19 (6.2)	286 (93.8)	
Pediatrician	136 (21.6)^¶^	493 (78.4)**		249 (39.6)	380 (60.4)		283 (45.0)	346 (55.0)		20 (3.2)	609 (96.8)	
Physician assistant	75 (21.2)^¶^	279 (78.8)**		50 (14.1)^¶^	304 (85.9)**		215 (60.7)**	139 (39.3)^¶^		34 (9.6) **	320 (90.4)^¶^	
**Gender^††^**												
Female	418 (27.5)	1,100 (72.5)	<0.001^§,§§^	468 (30.8)^¶^	1,050 (69.2)**	<0.001^§^	748 (49.3)**	770 (50.7)^¶^	<0.001^§^	74 (4.9)	1,444 (95.1)	0.082^§§^
Male	779 (36.8)	1,336 (63.2)		875 (41.4)**	1,240 (58.6)^¶^		737 (34.8)^¶^	1,378 (65.2)**		73 (3.5)	2,042 (96.5)	
Other	6 (42.9)	8 (57.1)		6 (42.9)	8 (57.1)		5 (35.7)	9 (64.3)		0 (—)	14 (100.0)	
**Age group, yrs**												
25–40	458 (34.5)	870 (65.5)	0.999	422 (31.8)^¶^	906 (68.2)**	0.001^§^	594 (44.7)**	734 (55.3)^¶^	0.019^§^	60 (4.5)	1,268 (95.5)	0.999
41–55	482 (32.1)	1,018 (67.9)		613 (40.9)**	887 (59.1)^¶^		561 (37.4)	939 (62.6)		59 (3.9)	1,441 (96.1)	
>55	263 (32.1)	556 (67.9)		314 (38.3)	505 (61.7)		335 (40.9)	484 (59.1)		28 (3.4)	791 (96.6)	
**Yrs in practice**												
3–10	446 (34.5)	845 (65.5)	0.999	424 (32.8)^¶^	867 (67.2)**	0.012^§^	572 (44.3)	719 (55.7)	0.006^§^	54 (4.2)	1,237 (95.8)	0.999
11–25	558 (32.3)	1,169 (67.7)		692 (40.1)**	1,035 (59.9)^¶^		643 (37.2)^¶^	1,084 (62.8)**		75 (4.3)	1,652 (95.7)	
>25	199 (31.6)	430 (68.4)		233 (37.0)	396 (63.0)		275 (43.7)	354 (56.3)		18 (2.9)	611 (97.1)	
**U.S. Census Bureau region** ^¶¶^												
Northeast	252 (32.3)	528 (67.7)	0.110	308 (39.5)	472 (60.5)	<0.001^§^	311 (39.9)	469 (60.1)	<0.001^§^	28 (3.6)	752 (96.4)	0.999
Midwest	297 (31.0)	662 (69.0)		322 (33.6)	637 (66.4)		425 (44.3)	534 (55.7)		35 (3.6)	924 (96.4)	
South	354 (31.3)	777 (68.7)		380 (33.6)	751 (66.4)		505 (44.7)	626 (55.3)		53 (4.7)	1,078 (95.3)	
West	300 (38.6)	477 (61.4)		339 (43.6)**	438 (56.4)^¶^		249 (32.0)^¶^	528 (68.0)**		31 (4.0)	746 (96.0)	
Urban-rural status***												
Urban	547 (39.4)**	840 (60.6)^¶^	<0.001^§^	573 (41.3)**	814 (58.7)^¶^	<0.001^§^	456 (32.9)^¶^	931 (67.1)**	<0.001^§^	66 (4.8)	1,321 (95.2)	0.999
Suburban	547 (29.3)^¶^	1,321 (70.7)**		680 (36.4)	1,188 (63.6)		813 (43.5)	1,055 (56.5)		69 (3.7)	1,799 (96.3)	
Rural	109 (27.8)	283 (72.2)		96 (24.5)^¶^	296 (75.5)**		221 (56.4)**	171 (43.6)^¶^		12 (3.1)	380 (96.9)	
**Work setting**												
Individual outpatient practice	196 (34.2)	377 (65.8)	0.007^§^	190 (33.2)	383 (66.8)	0.999	235 (41.0)	338 (59.0)	0.277	26 (4.5)	547 (95.5)	0.060
Group outpatient practice	754 (31.0)^¶^	1,682 (69.0)**		933 (38.3)	1,503 (61.7)		1,030 (42.3)	1,406 (57.7)		80 (3.3)	2,356 (96.7)	
Inpatient practice	253 (39.7)**	385 (60.3)^¶^		226 (35.4)	412 (64.6)		225 (35.3)	413 (64.7)		41 (6.4)	597 (93.6)	
**Approximate patient household income**												
<$25,000	79 (33.8)	155 (66.2)	0.999	72 (30.8)	162 (69.2)	0.999	102 (43.6)	132 (56.4)	0.999	9 (3.8)	225 (96.2)	0.870
$25,000–$49,999	300 (33.5)	595 (66.5)		323 (36.1)	572 (63.9)		378 (42.2)	517 (57.8)		31 (3.5)	864 (96.5)	
$50,000–$99,999	451 (31.4)	985 (68.6)		533 (37.1)	903 (62.9)		583 (40.6)	853 (59.4)		75 (5.2)	1,361 (94.8)	
$100,000–$249,999	251 (33.5)	498 (66.5)		276 (36.8)	473 (63.2)		315 (42.1)	434 (57.9)		27 (3.6)	722 (96.4)	
≥$250,000	122 (36.6)	211 (63.4)		145 (43.5)	188 (56.5)		112 (33.6)	221 (66.4)		5 (1.5)	328 (98.5)	

**Abbreviations**: 3HP = 3 months of once-weekly isoniazid plus rifapentine; 3HR = 3 months of daily isoniazid plus rifampin; 4R = 4 months of daily rifampin; 6H = 6 months of daily or twice weekly isoniazid; 9H = 9 months of daily or twice weekly isoniazid; LTBI = latent tuberculosis infection.

* Respondents could select more than one response. Respondents were asked to select one response to the question, “Do you routinely test non–U.S.-born patients for tuberculosis (TB)?” Those who selected “Prefer not to answer” were removed from the DocStyles sample (47). Percentages might not sum to 100 because of rounding.

^†^ Associations between provider characteristics and selection or nonselection of response options for LTBI testing variable were calculated using Pearson’s chi-square tests with Bonferroni-corrected p-values unless otherwise indicated, in which case Fisher’s exact test was used.

^§^ Value is statistically significant at p<0.05.

^¶^ Adjusted standardized residual ≤−3.6, indicating significantly less than expected cell value.

** Adjusted standardized residual ≥3.6, indicating significantly greater than expected cell value.

^††^ The “Other” option for gender was not available in the 2020 survey.

^§§^ Because of small cell sizes, significance was calculated using the Monte Carlo approximation for Fisher’s exact test (based on 10,000 sampled tables at a 99% CI). Adjusted standardized residuals were not calculated.

^¶¶^ https://www2.census.gov/geo/pdfs/maps-data/maps/reference/us_regdiv.pdf

*** Determined by the question, “How would you describe the community where you primarily work?”

## Discussion

CDC and the U.S. Preventive Services Task Force recommend testing persons at increased risk for TB infection as part of routine health care (*3*). HCPs are encouraged to use TB blood tests to test for TB infection; however, blood tests might not be available to all HCPs (*3*). It is not generally recommended to use both a TB skin test and a TB blood test to test the same person (*3*). Although recommended short-course LTBI treatment regimens are effective, safe, and associated with higher completion rates than are longer regimens (*8*), more HCPs indicated that they prescribe longer regimens or refer patients to a health department, with only one third reporting prescribing short-course regimens. The reasons for this were not identified in the survey; however, because of limited supplies of recommended drugs and intermittent shortages (*9*), short-course regimens might not be available at all times for all HCPs. Because not all health departments provide LTBI treatment or have the capacity to manage LTBI patients, future work is needed to identify barriers and implement interventions to facilitate prescribing LTBI treatment and managing LTBI patients by primary care providers.

Overall, U.S. TB case rates have declined during the past 2 decades (*2*); however, this trend could stagnate if actions to prevent TB are not implemented by HCPs serving groups experiencing disproportionate risk for TB and progressing from LTBI to TB disease. Because of gaps in provider knowledge and practice identified in this analysis of DocStyles results, priorities include continuing medical education about TB testing and LTBI treatment, especially among physician assistants and nurse practitioners; implementing interventions to improve HCP adherence to recommended practices (e.g., electronic medical record prompts); and identifying provider groups that might need resources to overcome barriers to implementing recommended TB testing and LTBI treatment.

### Limitations

The findings in this report are subject to at least four limitations. First, surveys relied on self-reported data, which are subject to recall and social desirability biases. Second, because participant characteristics might differ from the overall U.S. HCP population, results are not generalizable. Third, closed-ended survey questions did not allow for nuance in response. Finally, HCPs’ reasons for LTBI testing and treatment practices were not collected. Despite these limitations, DocStyles surveys provide valuable insights and can help guide outreach, education, and training efforts.

### Implications for Public Health Practice

CDC and partners provide resources^¶^ for providers on recommended practices for testing and treating TB and LTBI. To eliminate TB in the United States, further efforts are needed to address barriers for providers to test for and treat persons at risk for TB.

## Footnotes

*
https://www.cdc.gov/tb/statistics/reports/2021/table11.htm

¶
https://www.cdc.gov/tb/education/provider_edmaterials.htm
